# Orofacial clinical features in Arnold Chiari type I malformation: A case series

**DOI:** 10.4317/jced.54419

**Published:** 2018-04-01

**Authors:** José-Alcides de Arruda, Eugênia Figueiredo, João-Luiz Monteiro, Livia-Mirelle Barbosa, Cleomar Rodrigues, Belmiro Vasconcelos

**Affiliations:** 1 Department of Oral Surgery and Pathology, School of Dentistry, Universidade Federal de Minas Gerais, Belo Horizonte, MG, Brazil; 2 Hospital da Restauração, Universidade de Pernambuco, Recife, PE, Brazil; 3 Department of Oral and Maxillofacial Surgery, School of Dentistry, Universidade de Pernambuco, Camaragibe, PE, Brazil; 4 Department of Maxillofacial Prosthesis and Surgery, School of Dentistry, Universidade Federal de Pernambuco, Recife, PE, Brazil; 5 Department of Oral Imaging, School of Dentistry, Faculdades Integradas da União Educacional do Planalto Central, Brasília, DF, Brazil

## Abstract

**Background:**

Arnold Chiari malformation (ACM) is characterized by an anatomical defect at the base of the skull where the cerebellum and the spinal cord herniate through the foramen magnum into the cervical spinal canal. Among the subtypes of the condition, ACM type I (ACM-I) is particularly outstanding because of the severity of symptoms. This study aimed to analyze the orofacial clinical manifestations of patients with ACM-I, and discuss their demographic distribution and clinical features in light of the literature.

**Material and Methods:**

A case series with patients with ACM-I treated between 2012 and 2015 was described. The sample consisted of patients who were referred by the Department of Neurosurgery to the Oral and Maxillofacial Surgery Service of Hospital da Restauração in Brazil for the assessment of facial symptomatology. A questionnaire was applied to evaluate the presence of painful orofacial findings. Data are reported using descriptive statistical methods.

**Results:**

Mean patient age was 39.3 years and the sample consisted mostly of male patients. A high prevalence of headache (50%) and pain in the neck (66.7%) and masticatory muscles (50%) was found. Only one patient reported difficulty in performing mandibular movements and two reported jaw clicking sounds. Mean mouth opening was 40.83 mm.

**Conclusions:**

ACM-I patients may exhibit orofacial symptoms which may mimic temporomandibular joint disorders. This study brings interesting information that could help clinicians and oral and maxillofacial surgeons to understand this uncommon condition and also help with the diagnosis of patients with similar physical characteristics by referring them to a neurosurgeon.

** Key words:**Arnold-Chiari malformation, facial pain, diagnosis, orofacial.

## Introduction

Arnold Chiari malformation (ACM) was first described by Cleland in 1883 and later described in detail by the same author in 1891 (1). ACM belongs to a group of congenital conditions characterized by an anatomic defect of the base of the skull, in which the cerebellum and brain stem herniate through the foramen magnum into the cervical spinal canal (2,3). To date, there is no consensus regarding the etiology of ACM, and several theories have been proposed for its pathogenesis (4). It is believed to be due to a defect involving the sclerotomes during the development of the occipital bone, resulting in a posterior cranial fossa that is too small to accommodate the cerebellum (5).

Among the subtypes of ACM, Arnold Chiari malformation type I (ACM-I) is particularly outstanding due to the severity of its symptoms. The diagnosis can be made during childhood, with an incidence of 1:1,000-5,000 (6,7), although the signs and symptoms of the condition may appear from the fourth to the sixth decade of life. Due to an often late diagnosis of the condition, oral and maxillofacial information about it is scarce. Women are more frequently affected (8,9), with the condition being accompanied by sensorimotor and autonomic manifestations (10).

Data from an American Institute have shown that many patients with ACM remain asymptomatic and do not require neurosurgical intervention (11). Langridge *et al.* (8) stated that most asymptomatic persons with ACM-I (93.3%) remained asymptomatic even in the presence of syringomyelia, which is a small dilatation of the central spinal canal extending through a few spinal segments frequently associated with ACM-I (12). The most frequent clinical findings observed in ACM-I patients are suboccipital headache, weakness in the extremities, facial numbness, loss of temperature sensation, ataxia, diplopia, dysarthria, dysphagia, vomiting, vertigo, nystagmus, and tinnitus (13). Approximately 20% of patients with ACM-I may have dysfunctions such as trigeminal or glossopharyngeal neuralgia, paralysis of the vocal cords, hoarseness, sleep apnea, crichopharyngeal achalasia, soft palate weakness, tongue atrophy, and facial hypesthesia (7,14-16).

Pain along the course of the trigeminal nerve may be associated with changes in intracranial pressure and may affect the trigeminal tract that runs dorsolaterally to the spinal cord (2,17), consequently mimicking the signs and symptoms of temporomandibular joint disorder (TMD). The various clinical problems that affect the masticatory muscles and the temporomandibular joint (TMJ) may be concomitantly associated with a primary disease or disorder (18,19). The usual treatment is expansion of the foramen magnum by decompression of the posterior cranial fossa in combination or not with other surgical techniques (8). Within this context, the aim of the present study was to analyze the orofacial clinical findings of a case series of ACM-I patients treated at a referral Hospital in the Brazilian Northeast.

## Material and Methods

-Study design, ethical issues and sample

A case series was analyzed in the present study, involving ACM-I patients of both sexes and of different ages. The sample consisted of patients referred by the Department of Neurosurgery to the Oral and Maxillofacial Surgery Service of Hospital da Restauração, Recife (PE, Brazil) from 2012 to 2015 for assessment of facial signs and symptoms. The study was approved by the Ethics Committee of Hospital da Restauração (Approval No. 48101415.2.0000.5198), and the authors followed the guidelines of the Helsinki Declaration.

Data were collected using the Fonseca Anamnestic Index (20), which contains objective questions related to the orofacial complex. It is a low cost and easy to apply alternative for screening TMD patients. In Brazil, Fonseca’s Anamnestic Index has frequently been used. This index is affordable, quick, and easy to apply questionnaire with high accuracy, sensitivity, and specificity for evaluating individuals with TMD. Gender and age were obtained, and the data were grouped. Patients with psychiatric diseases associated with ACM-I were excluded.

-Data analysis

Descriptive and quantitative data analysis was performed using the Statistical Package for the Social Sciences (SPSS) software, version 22.0 (SPSS Inc., Chicago, IL, USA).

## Results

**Figure 1:**
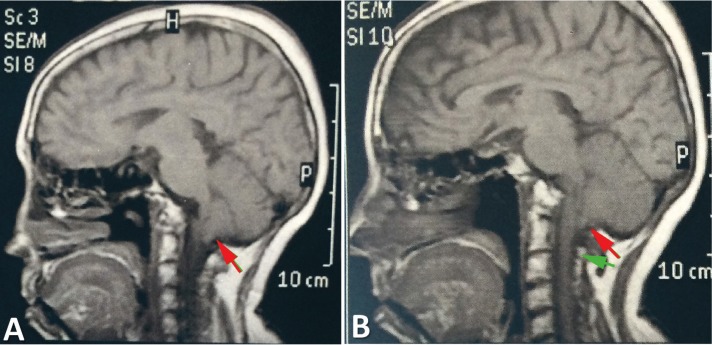
Sagittal magnetic resonance reconstructions (A and B) demonstrate part of the cerebellum entering the vertebral canal (red arrow) and positioning itself posterior to the spinal cord. Cerebrospinal fluid (green arrow).

**Figure 2:**
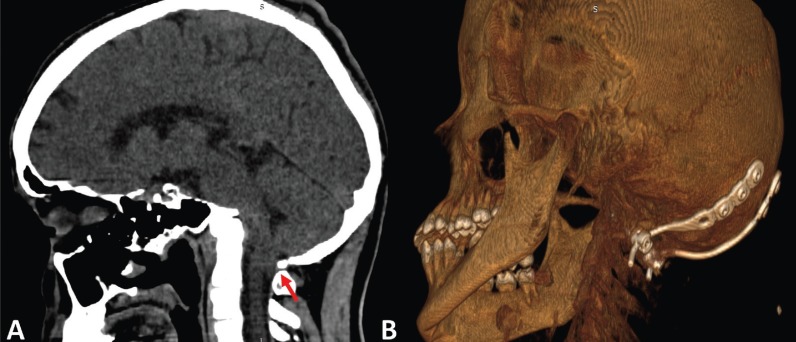
ACM-I male patient. A. The computed tomography (CT) scan demonstrates the cerebellum herniation (red arrow). B. Post-operative CT scan of the same patient. Stabilization of the cervical vertebrae was also required (arthrodesis).

**Figure 3:**
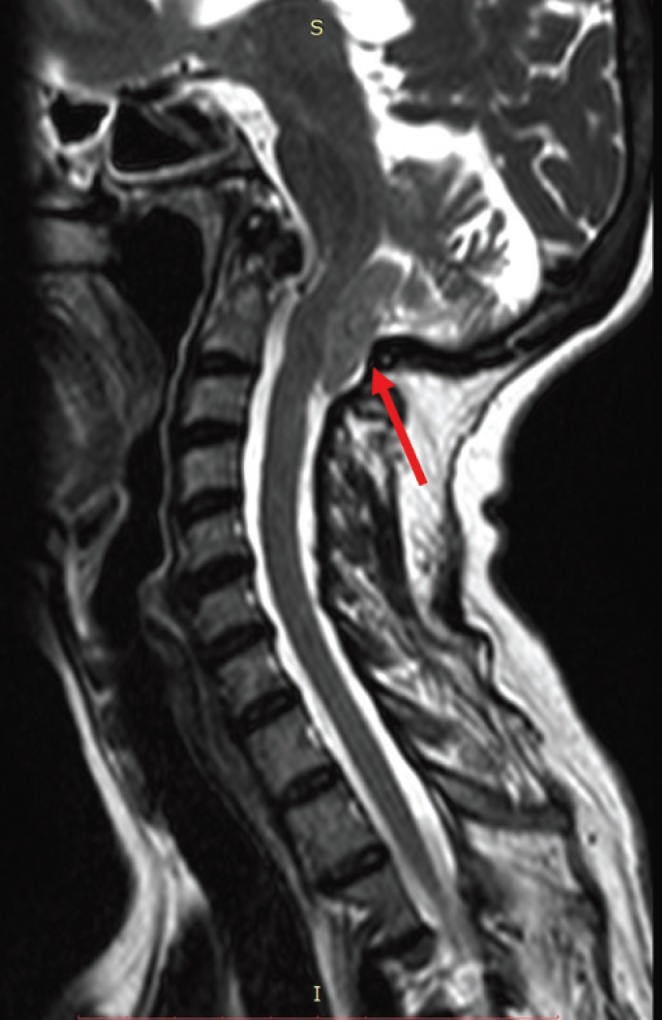
Sagittal magnetic resonance reconstructions of an ACM-I female patient demonstrating the cerebellum herniation into the vertebral canal (red arrow).

The study was conducted on six patients (four men and two women) ranging in age from 20 to 62 years (mean: 39.3 years). The imaginological features of ACM-I are shown in figures 1, 2, and 3.

 Table 1 presents the data related to headache, painful symptoms in the TMJ and mandibular movements. Three patients reported difficulty or pain, or both, in opening and/or closing the mouth, with the problem only arising sporadically in two of them. Two patients reported difficulty in moving the jaw forward or sideways, with this problem occurring only at times in one of them. Three patients stated that they felt tired or experienced muscle pain when chewing. Five patients reported headache, four reported pain in the neck region and two reported earache or pain in regions close to the ears.

**Table 1:**
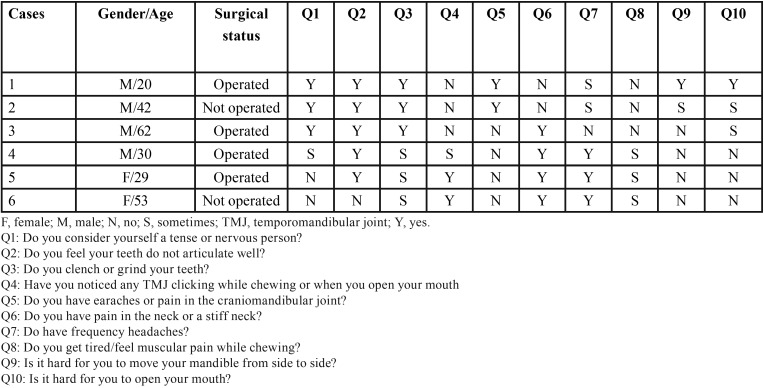
Orofacial findings of patients with Arnold Chiari malformation type I.

Four of the six patients had been operated upon by the neurosurgery team of the Hospital for expansion of the foramen magnum and decompression of the posterior cranial fossa in order to correct herniation of the cerebellum along the spinal cord.

Mean mouth opening measured by the distance between upper and lower incisors was 40.83 mm.

## Discussion

The objective of the present study was to determine the presence of orofacial findings in patients with ACM-I and to emphasize the importance of a differential diagnosis in the assessment of patients with painful symptoms. Several studies have demonstrated that patients with symptoms similar to those observed in TMD exhibited another comorbidity, explaining why many patients submitted to various treatments continued to have chronic pain. Thus, it is important for clinicians who examine patients with painful symptoms in the orofacial region to consider any comorbidity (19).

ACM-I is defined as a cerebellar herniation of 3 to 5 mm below the level of the foramen magnum (21), with neurological dysfunctions probably due to compression of nervous tissue in the craniovertebral junction (22). Although most cases are congenital, etiology of the condition has been reported after trauma or tumors, with ACM-I possibly showing evolutionary characteristics at any age, although normally during youth (5). Some studies have reported that about 30% of ACM-I cases are asymptomatic (4,23). In the present sample, all patients had painful symptoms related to the orofacial structures.

The results of the present study should be interpreted with caution. Symptoms similar to those detected in TMD were present in all patients, with emphasis on pain of the masticatory muscles, fatigue when chewing, and headache. The high prevalence of orofacial findings in the present study was not sufficient to diagnose these patients as having TMD. Indices and criteria helping with the diagnosis of TMDs are currently available, such as the DC/TMD which, in addition to assessing pain and parafunction, also looks for other aspects such as depression and anxiety (25). However, in the present study we opted to use the Fonseca Anamnestic Index (20) because it contains objective questions related to the orofacial structures. It is important to point out the good mean mouth opening of the sample (40.83 mm). Only one patient reported difficulty in performing mandibular movements, and two reported clicking noises in the TMJ region, indicating that these patients were unlikely to have some change localized in the joint, but rather that the facial symptoms were probably due to the underlying neurological disease, suggesting a central etiology of the pain. However, studies conducted on larger samples at referral centers with a more detailed investigation related to the TMJ and associated structures are needed, since disorders of the joints may coexist, possibly being amplified in these patients.

Peñarrocha *et al.* (26) reported the case of a patient with orofacial pain for whom causes related to dental focal points and to changes in the TMJ were first investigated. Dental treatment was performed but the patient did not report clinical improvement. Thus, ACM was detected after an accurate imaging investigation and neurosurgical treatment, resulting in improved orofacial symptoms. On this basis, patients with this disorder at times may be underdiagnosed, underscoring the importance of awareness of this malformation on the part of clinicians, general dentists and oral and maxillofacial surgeons. Even though the condition is unusual, both a clinical and imaging differential diagnosis should be established since patients with ACM may report symptoms in the dental arches as well as the TMJ and irradiation towards the oropharynx and the ears and adjacent nerves (10,27,28), which does not exactly indicate the presence of TMD.

There is no consensus about the prevalence of this disease and predilection for the female gender has been discussed in the literature (8). In the present study, men were more affected. However, the estimated presence of ACM is low, ranging from 0.1% to 0.5%.

Few studies have reported oral or maxillofacial findings for patients with ACM-I and have correlated them to symptoms similar to those detected in TMDs. The diagnosis of ACM-I is a challenge and is usually made by a neurosurgeon. Orofacial pain may not correlate with the maxillofacial image findings in patients with ACM-I (27,28). The diagnosis is usually made with the aid of magnetic resonance of the cerebellum.

The treatment of ACM consists of improving the region of the foramen magnum in the craniovertebral junction in an attempt to reduce the painful symptoms by reducing the nerve compression caused by cerebellar herniation (8). Although there are no unanimous criteria about surgical intervention, with a rate ranging from 9 to 75%, Victorio and Khoury (28) have stated that there is a consensus about considering the possibility of surgical intervention for ACM when the clinical and imaging signs and symptoms are clearly correlated. The surgical diagnosis is variable (8). In the present sample, some patients had undergone surgery for expansion of the foramen magnums in order to decompress the posterior cranial fossa. These patients should be followed up for a long time and a multidisciplinary team is required for their management.

## Conclusions

In summary, the present patients with ACM-I exhibited orofacial findings. More studies on larger samples are required in order to analyze the orofacial findings correlated with the TMJ. Moreover, this study brings interesting information that could help clinicians and oral and maxillofacial surgeons with the diagnosis and management of this condition.
